# B Cell-Based Seamless Engineering of Antibody Fc Domains

**DOI:** 10.1371/journal.pone.0167232

**Published:** 2016-12-01

**Authors:** Koji Hashimoto, Kohei Kurosawa, Akiho Murayama, Hidetaka Seo, Kunihiro Ohta

**Affiliations:** Department of Life Sciences, Graduate School of Arts and Sciences, The University of Tokyo, Tokyo, Japan; University of British Columbia, CANADA

## Abstract

Engineering of monoclonal antibodies (mAbs) enables us to obtain mAbs with additional functions. In particular, modifications in antibody’s Fc (fragment, crystallizable) region can provide multiple benefits such as added toxicity by drug conjugation, higher affinity to Fc receptors on immunocytes, or the addition of functional modules. However, the generation of recombinant antibodies requires multiple laborious bioengineering steps. We previously developed a technology that enables rapid *in vitro* screening and isolation of specific mAb-expressing cells from the libraries constructed with chicken B-cell line DT40 (referred to as the ‘ADLib system’). To upgrade this ADLib system with the ability to generate customized mAbs, we developed a novel and rapid engineering technology that enables seamless exchanges of mAbs’ Fc domains after initial selections of mAb-producing clones by the ADLib system, using a gene-replacement unit for recombinase-mediated cassette exchange (RMCE). In this system, Cre-recombinase recognition sites were inserted into the Fc region of the active DT40 IgM allele, allowing the replacement of the Fc domain by the sequences of interest upon co-transfection of a Cre recombinase and a donor DNA, enabling the rapid exchange of Fc regions. Combining this method with the ADLib system, we demonstrate rapid Fc engineering to generate fluorescent antibodies and to enhance affinity to Fc receptors.

## Introduction

Antibodies (Ab), also known as immunoglobulins (Ig), have been widely used for therapeutic, diagnostic and research purposes [1]. Particularly, monoclonal antibodies (mAbs), which specifically bind to a given antigen, are valuable as drugs or research reagents due to their superior homogeneity. Among several methods for mAb generation, the hybridoma method [2] is the most successful one, although there is room for improvement. For instance, it is difficult to generate mAbs against poorly immunogenic antigens such as auto-antigens, toxic compounds and lipids. Moreover, it consists of time-consuming steps such as animal immunization, while *in vitro* screening systems like phage display can overcome these disadvantages [3]. However, phage display has its weakness in the time-consuming recombinant DNA engineering steps, which may take up to several weeks [4]. In addition, the specificity of scFv antibodies converted from phage antibodies is sometimes reduced or altered when transferred to the full length antibody format [5].

We previously developed an *in vitro* method to obtain mAbs using the chicken B cell-derived DT40 cell line that expresses both membrane-bound and secreted forms of IgM antibodies [6,7]. This technology, named the ADLib system (Autonomously Diversifying Library system), enables rapid generation of antigen-specific mAbs (within about 1 week) from animal-free libraries. ADLib-generated mAbs have been successfully used for various applications, such as ELISA, flow cytometry and immunofluorescence microscopy [6–10].

Antibodies are Y-shaped proteins that consist of two parts [11]: the variable (V) region and the constant (C) region. The V region is genetically diverse to enable potential immunity against a wide variety of antigens. Unlike human or mouse which possess multiple V genes, the chicken immunoglobulin locus contains only one functional V gene downstream of a cluster of pseudo V genes [12]. The sole functional V gene in chicken is diversified not by V(D)J recombination but by gene conversion, a type of homologous recombination in which part of the functional V gene is iteratively overwritten by the pseudo V gene segments [13–15]. In DT40 cells, low-frequency gene conversion occurs at the V region of the immunoglobulin locus [16,17]. We discovered that treatment of DT40 cells with a histone deacetylase inhibitor, trichostatin A (TSA), enhances the frequency of gene conversion at the V region [6,18]. The ADLib system exploits TSA-dependent enhancement of gene conversion to prepare autonomously expanding libraries of antibody-expressing cells, and specific mAbs against user-defined antigens can be isolated from the libraries [6,7].

The other part of the antibody molecule referred to as C (constant) region, which is genetically constant and carries the Fc domain, determines antibody isotype. The C region is switchable between different isotypes by class switch recombination (CSR) [19]. Each antibody isotype binds to a different receptor and plays distinct roles in the immune system [20–22]. The binding of Fc receptors to their respective Fc domain depends upon the structure of the Fc region. To date, multiple studies have been published focusing on Fc region optimization to generate more effective mAbs as therapeutic drugs or reagents for biological research [23,24].

While the ADLib system enables rapid *de novo* generation of mAbs *in vitro*, one obvious caveat is that DT40 cells express chicken IgMs [25]. Generally, due to its instability and low yield of purification, IgM-type antibodies are less preferred than IgG, which is widely used for therapeutic, diagnostic and research purposes [26]. In our previous paper, we have successfully developed a DT40 cell line expressing chicken/human chimeric IgG antibody and applied it to the ADLib system [27]. In this chimeric ADLib system, the coding sequence of the human IgG1-Fc (hIgG1-Fc) region was integrated into the chicken IgM heavy-chain locus by gene targeting. The resulting transfectants expressed chicken IgM and chicken/human chimeric IgG simultaneously. Chicken IgM and chimeric IgG exhibited similar affinity to the antigen because these two antibodies shared identical antigen-binding regions. By this approach, we can isolate monoclonal chimeric IgGs from the antibody library without any further gene manipulation steps after antibody selection. Meanwhile the success rate of gene targeting at the chicken IgM heavy-chain locus was much lower (0.77%) than those of other gene loci (about 50%) possibly due to large tandem repeats in the coding sequence of chicken IgM-Fc [25,27–29].

**Fig 1:**
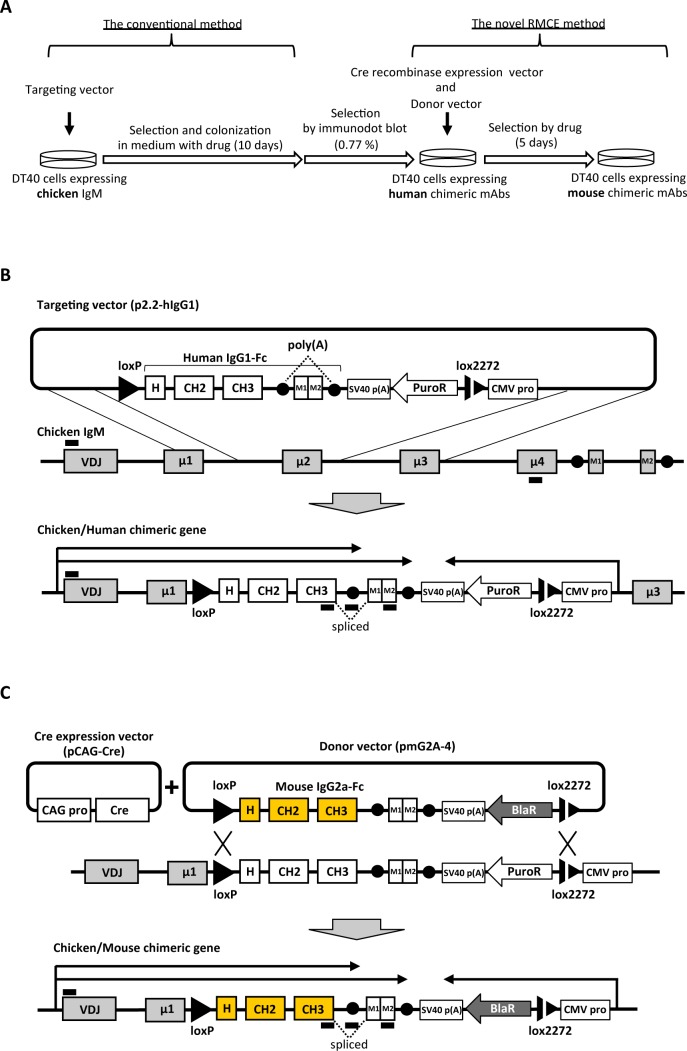
Scheme for development of RMCE host cell lines and Fc cassette replacement by RMCE. A) Experimental outline for generating chimeric mAbs. The endogenous chicken IgM-Fc region was replaced with hIgG1-Fc cassette by the conventional gene targeting method. HIgG1-Fc was switched to mIgG2a-Fc by RMCE. B) Scheme for development of RMCE host cell lines. The targeting vector (p2.2-hIgG1) was designed so that it targets the chicken IgM Fc region at Ig heavy chain locus. P2.2-hIgG1 contains hIgG1-Fc and puromycin resistance gene in the opposite orientation. LoxP and lox2272 were placed upstream of hIgG1-Fc and between CMV promoter and cDNA of puromycin resistance gene of p2.2-hIgG1, respectively. The transmembrane domains of chicken IgM (M1 and M2) and a polyA signal were inserted downstream of hIgG1-Fc and its polyA in order to simultaneously express both membrane-bound type and secreted types of chimeric antibodies by alternative splicing. Black bars below the gene structure diagram indicate the positions of RT-PCR primers for Fig 2C. C) Scheme for the RMCE strategy. Cre recombinase expression vector (pCAG-Cre) and the donor vector (pmG2A-4) including the mIgG2a-Fc gene and a promoter-less blasticidin S resistance gene were co-transfected, and DT40 cells properly replaced by its hIgG1-Fc with mIgG2a-Fc were selected by addition of blasticidin S. Black bars below the gene structure diagram indicate the positions of RT-PCR primers for Fig 2G.

In this study, we utilized recombinase-mediated cassette exchange (RMCE) based on the Cre/loxP system [30] to overcome the difficulty in gene manipulation at this specific locus. We achieved this by first integrating specific loxP sites to the Fc region of wild-type DT40 cells. Although the success rate of this first step is low, as mentioned above, this enables the Fc region to be easily exchanged to any other Fc of interest by RMCE. This strategy combines gene targeting and RMCE, allowing dramatic improvement of the efficiency of gene manipulation at the Fc region. Cre recombinase recognizes a specific sequence called loxP to catalyze DNA recombination between flanking loxP sites [31]. Furthermore, the loxP mutant 2272 has been shown to recombine with an identical mutant, but not with the wild-type loxP sequence. This feature indicates that the combination of wild type loxP and lox2272 is suitable for cassette replacement [32]. The RMCE-based strategy for Fc exchange presented here (Fig 1A) is composed of two steps. First, hIgG1-Fc flanked by loxP and lox2272 is inserted by knock-in to the Fc region of DT40 (Fig 1B). Second is RMCE-based switching of hIgG1-Fc to any other desired sequence flanked by loxP and lox2272, in our example mouse Fc (Fig 1C). With this new strategy, any Fc sequence of interest can be integrated to DT40 antibodies, either before or after isolation of specific mAbs by the ADLib system. We demonstrate the usefulness of this method by generating a fluorescent antibody and optimizing antibody-Fc receptor interactions.

## Results

### Development of RMCE host cell line by integrating the human IgG-Fc cassette flanked by loxP and lox2272 into the endogenous chicken IgM-Fc region

DT40 cells express secreted and membrane-bound forms of IgM [25,33]. The constant region is composed of four exons (Cμ1, Cμ2, Cμ3, Cμ4), and the Fc region is composed of three exons (Cμ2, Cμ3, Cμ4) (Cμ1 associates with Ig light chain) [29,34]. We developed a DT40 cell line expressing chicken/human chimeric IgG1 by integrating the coding sequence of hIgG1-Fc into the genomic region between exon Cμ1 and Cμ3 by disrupting Cμ2. The targeting vector named p2.2-hIgG1, carries a puromycin resistance gene (puroR) under the control of the cytomegalovirus (CMV) promoter in the opposite orientation from hIgG1-Fc, and the loxP and lox2272 sites were integrated upstream of the hIgG1-Fc and between puroR and its CMV promoter, respectively (Fig 1B). The vector also has two homologous sequences for gene targeting, and was transfected with a conventional method as described previously [27]. After transfection, colonies were prepared from single cells in 96-well plates containing medium supplemented with puromycin, followed by screening for properly targeted clones by immunodot blot analysis of culture supernatants using anti-hIgG-Fc antibody (Fig 1A).

**Fig 2:**
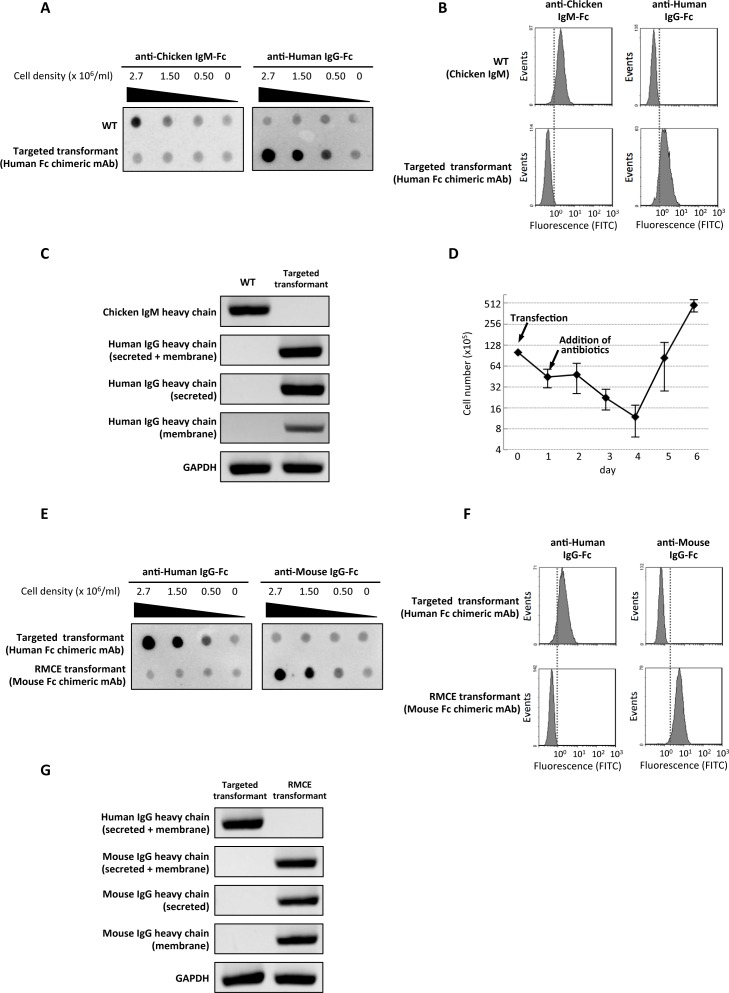
Expression of chimeric antibodies in transformed DT40 cells. A) Immunodot blot with HRP-conjugated anti-Chicken IgM-Fc (left) and anti-Human IgG-Fc (right). Wild-type DT40 (WT) and targeted transformants (Human Fc chimeric mAb) were diluted at various cell densities and cultured for 48 hours. The equal volumes of culture supernatants were spotted onto the membrane. The final cell density is indicated above each dot blot image. B) Flow cytometry analysis with FITC-conjugated anti-Chicken IgM-Fc (upper left and lower left) and anti-Human IgG-Fc (upper right and lower right). Wild-type DT40 (WT: upper left and upper right) and targeted transformants (lower left and lower right) were analyzed. C) RT-PCR analysis to examine the expression of chicken IgM and chicken/human chimeric IgG. The primer annealing positions were indicated by black bars in Fig 1B. RNA was extracted from wild-type DT40 (WT) and targeted transformants (Human Fc chimeric mAbs) expressing hIgG1. D) Monitoring of viable cell number after transfection followed by antibiotic selection. After RMCE-mediated exchange of hIgG1-Fc to IgG2a-Fc, blasticidin S was added to the medium and the number of living cells was counted every day for 6 days after the transfection. E) Immunodot blot with HRP-conjugated anti-Human IgG-Fc (left) and anti-Mouse IgG-Fc (right). The culture supernatants of targeted transformants (Human Fc chimeric mAbs) and the cells with the targeted transformants’ Fc region swapped with mIgG2a by RMCE (RMCE transformant (Mouse Fc chimeric mAb)) were prepared as described in Fig 2A. F) Flow cytometry analysis with FITC-conjugated anti-Human IgG-Fc (upper left and lower left) and anti-Mouse IgG-Fc (upper right and lower right). Targeted transformants (upper left and upper right) and RMCE transformants (lower left and lower right) were analyzed. G) RT-PCR analysis to examine the expression of human chimeric IgG and mouse chimeric IgG. The primer annealing positions were indicated by black bars in Fig 1C. RNA was extracted from targeted transformants (Human Fc chimeric mAbs) and RMCE transformants (Mouse Fc chimeric mAbs).

We analyzed 261 puromycin-resistant clones and could obtain two positive clones (S1 Fig). To confirm the disruption of Cμ2 and integration of the construct into the target locus, the cells were analyzed by immunodot blot, flow cytometry and RT-PCR. Every analysis revealed that the positive clone in the primary screening expresses chicken/human chimeric IgG, both in the secreted and membrane-bound forms, instead of chicken IgM (Fig 2A, 2B and 2C).

### Cassette replacement by RMCE

Next, to prove that the antibodies’ Fc regions can be switched easily to another sequence by RMCE, we attempted to replace the Fc regions of hIgG1 chimeric mAbs described above with those of murine IgG2a.

The donor vector and Cre recombinase expression vector were co-transfected to the RMCE host cell line that expresses chicken/human chimeric IgG. The donor vector named pmG2A-4 consists of the mouse IgG2a-Fc (mIgG2a-Fc) region and a promoter-less blasticidin S resistance marker (BlaR) flanked by loxP and lox2272 sites in 5’ and 3’, respectively (Fig 1C). 24 hours after the transfection, blasticidin S was added to the medium and after culturing for another four days, the number of the blasticidin S-resistant cells was almost identical to the initial number of cells before transfection (Fig 2D). To confirm the replacement of Fc region by RMCE, the cells selected by blasticidin S were analyzed by immunodot blot, flow cytometry and RT-PCR (Fig 2E, 2F and 2G). As expected, chicken/mouse chimeric IgGs were expressed in their secreted and membrane-bound forms, while the expression of chicken/human chimeric IgG had disappeared (Fig 2E and 2F). Flow cytometry analysis showed that the frequency of successful gene replacement by RMCE was more than 98% (Fig 2F). Even the mRNA coding chicken/human chimeric IgG was not detectable by RT-PCR (Fig 2G).

### Enhancement of chimeric IgG expression by insertion of BGH terminator

The amount of chimeric IgG on the cell surface of DT40 cells is an important factor for successful generation of specific mAbs by the ADLib system, since the antigen-specific clones are isolated by physical interaction between antigen-conjugated magnetic beads and membrane-presented antibodies [6]. Flow cytometry analysis suggested that the amount of membrane-bound chicken/human chimeric IgG in the targeted transformants is likely to be significantly lower than that of chicken IgM on the surface of wild type cells (Fig 2B), which may lead to reduced efficiency of the clonal selection when using the ADLib system.

**Fig 3:**
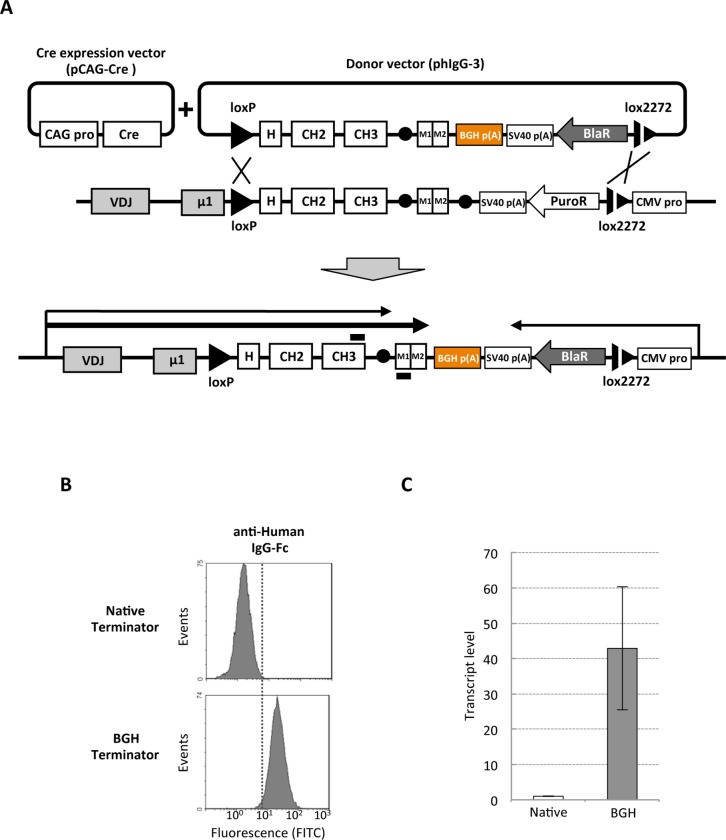
Enhancement of chimeric IgG expression by BGH terminator. A) Schematic view of the RMCE strategy to replace the native chicken IgM heavy chain terminator with BGH terminator. PhIgG-3 vector contains hIgG1-Fc, a promoter-less blasticidin S resistance gene and the BGH terminator. PhIgG-3 and pCAG-Cre were co-transfected, and the cells in which native terminator is replaced with BGH were selected by the addition of blasticidin S. B) Flow-cytometric analysis with FITC-conjugated anti-hIgG-Fc. The hIgG1 chimeric mAb expressing cells harboring native chicken IgM heavy chain terminator (Native Terminator) and those of which the terminator was replaced with the BGH terminator (BGH Terminator) by RMCE were analyzed. C) Quantitative RT-PCR analysis of the expression levels of membrane-bound hIgG chimeric mAbs. RNA was extracted from cells harboring the native terminator (Native Terminator) and those after RMCE-induced replacement with the BGH terminator (BGH Terminator). The transcript levels were normalized by GAPDH signals. The results are from two independent experiments. The primer annealing sites are indicated by black bars in Fig 3A.

To solve this problem, the native terminator downstream of the specific exons for membrane-bound form of hIgG1 was replaced by the stronger BGH (Bovine Growth Hormone) terminator by RMCE, which is known to stabilize and improve mRNA expression[35]. The vector named phIgG-3 consists of the coding sequences of hIgG1-Fc, two exons for the membrane-bound domain, the BGH terminator, and a promoter-less blasticidin S resistance gene flanked in 5’ and 3’ by loxP and lox2272 sites, respectively. This vector was co-transfected along with the Cre recombinase expression vector to DT40 cells expressing chicken/human chimeric IgG (Fig 3A). After selection by blasticidin S, the membrane-bound form IgG was analyzed by flow cytometry and quantitative RT-PCR. Compared to parental cells, the amount of membrane-bound IgG was significantly higher in the cells harboring BGH terminator (Fig 3B). Quantitative RT-PCR showed about 40-fold increase in the quantity of chimeric IgG mRNA after incorporating the BGH terminator (Fig 3C).

### Isolation of antigen-specific chimeric mAbs by the ADLib system followed by one-step Fc alteration by RMCE

**Fig 4:**
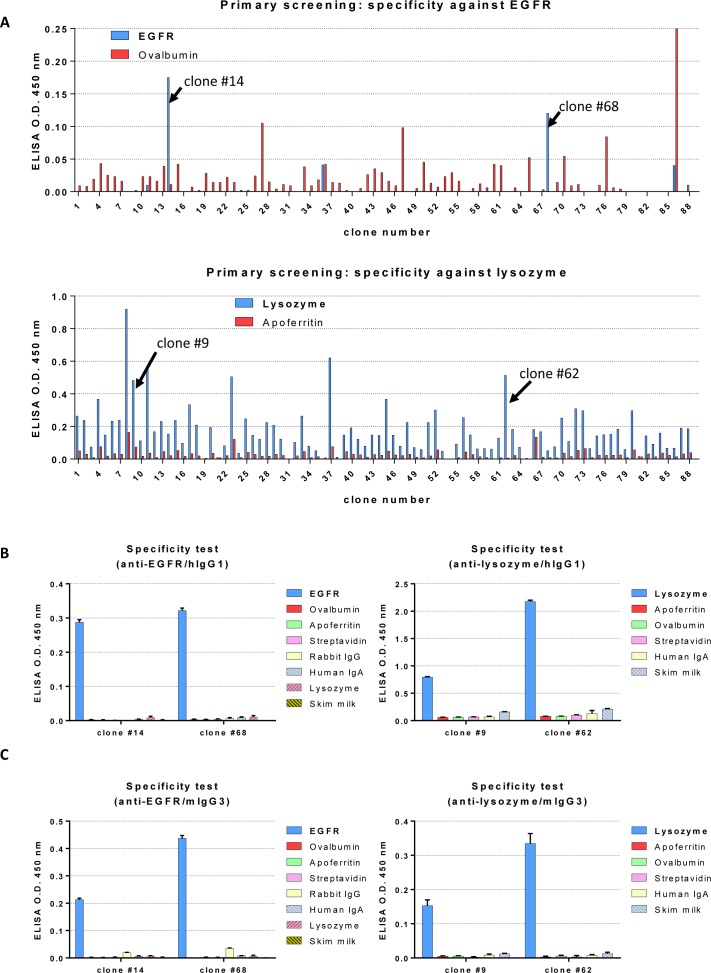
Isolation of antigen-specific chimeric mAbs by the ADLib system and Fc modification by RMCE. A) Screening of hIgG1 chimeric mAbs specific to EGFR or lysozyme by ELISA. Libraries constructed from hIgG1 chimeric mAb-expressing cells were prepared and subjected to the ADLib system by using magnetic beads conjugated with either EGFR or lysozyme. The culture supernatants of the selected clones were subjected to ELISA to examine their respective binding activities to EGFR (upper) and lysozyme (lower). Ovalbumin and apoferritin were used as negative controls. The clones indicated by the arrows were further analyzed in the experiment described in Fig 4B. B) ELISA analysis to confirm the specificities of mAbs selected by the ADLib system. (left) Specificities of anti-EGFR mAbs after primary screening. Ovalbumin, apoferritin, streptavidin, rabbit IgG, human IgA, lysozyme, and skim milk were used as negative controls. (right) Specificities of anti-lysozyme mAbs after primary screening. Apoferritin, ovalbumin, streptavidin, human IgA and skim milk were used as negative controls. C) ELISA analysis to confirm the specificities of the Fc-replaced mAbs by RMCE after selection by the ADLib system. The Fc region of the clones shown in Fig 4B was replaced with mIgG3. Specificities of anti-EGFR (left) and anti-lysozyme (right) mAbs were examined by ELISA. Negative control antigens are identical to those described in Fig 4B.

Using the BGH terminator-inserted clones, we attempted to isolate clones expressing antigen-specific IgG using the ADLib system. We first prepared diversified DT40 libraries by treating the cells with 2.5 ng/mL of TSA for four weeks. After TSA treatment, DT40 cells expressing diversified membrane-bound antibodies were subjected to a selection to isolate the antigen-specific clones by using magnetic beads coated with either the extracellular domain of epidermal growth factor receptor (EGFR) or lysozyme. We successfully isolated several anti-EGFR and anti-lysozyme clones (Fig 4A). Further analysis with ELISA also showed that the isolated clones have high specificity to the targets (Fig 4B).

To verify the applicability of one-step Fc alteration by RMCE after antibody isolation, we attempted to swap the hIgG1-Fc regions to mouse Fc regions. Two donor vectors, referred to as pmG3-4BP and pmG2A-4BP, which consist of mouse IgG3 and IgG2a Fc regions, respectively, along with a promoter-less puromycin resistance marker (PuroR), and loxP and lox2272 flanking sites, were alternatively co-transfected to the isolated antigen-positive cells with a Cre recombinase expression vector. After selection by puromycin, replacement of the Fc regions was confirmed by flow cytometry using anti-mIgG-Fc antibody (data not shown). To investigate the specific binding of mouse chimeric mAbs to their antigens, the culture supernatants were subjected to ELISA analysis. The results showed that the mIgG3 chimeric (Fig 4C) and mIgG2a chimeric mAbs (S2 Fig) retained a specificities comparable to those of the original antibodies.

The anti-EGFR and anti-lysozyme mAbs described above were first obtained in the form of hIgG1 chimeric mAbs followed by the replacement of their hIgG1-Fcs with mouse Fcs using the RMCE method. Meanwhile, another approach by which the Fc is exchanged first, prior to the library preparation is possible: 1) replacement of the Fc of hIgG1 chimeric mAb-expressing cells with mouse Fc by RMCE; 2) library construction with mouse chimeric mAb-expressing cells; 3) selection of antigen-specific mouse chimeric mAb-expressing clones. The anti-EGFR mIgG2a mAbs were generated successfully by both approaches (S2 and S3 Figs). These data suggest that the RMCE method enables the exchange of mAb’s Fc either before or after the isolation of mAbs.

### Biochemical properties of chimeric mAbs

**Fig 5:**
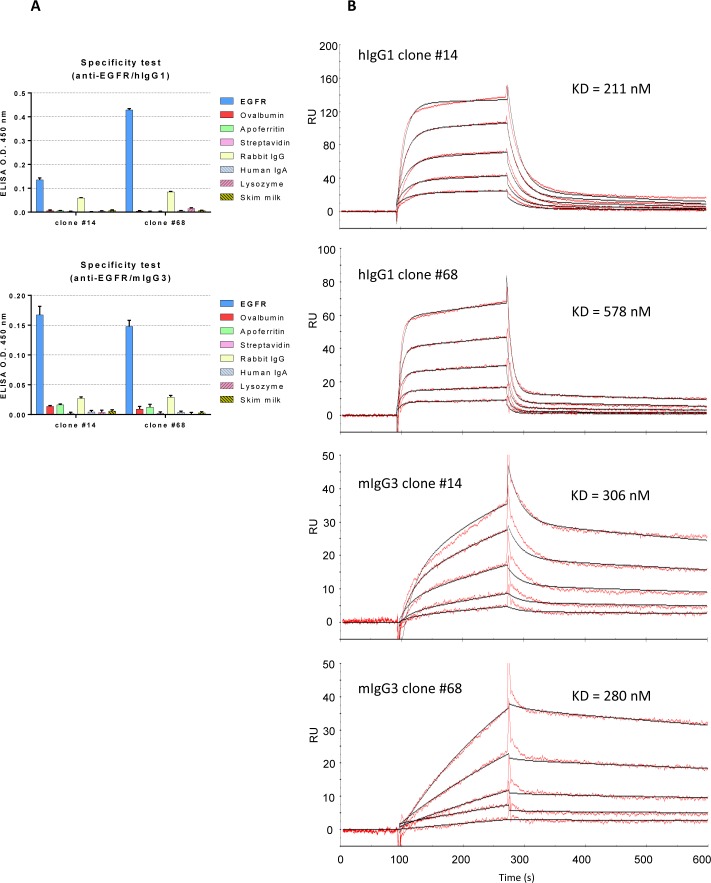
Specificities and binding affinities of purified chimeric antibodies. A) ELISA analysis to confirm the specificities of the purified hIgG1 chimeric (upper) and mIgG3 chimeric (lower) anti-EGFR mAbs. Negative control antigens are identical to those described in Fig 4B. B) SPR analysis of anti-EGFR chimeric mAbs. Serially diluted purified chimeric mAbs were applied to the EGFR-Fc immobilized biosensor chip. The KDs were calculated using a bivalent analyte model.

To examine the feasibility of this system for further practical applications, we purified the chimeric mAbs from the culture supernatant and assessed their binding properties. First, we optimized the culture conditions of cells expressing chimeric mAbs using serum-free medium to avoid contamination by IgGs derived from fetal bovine serum (FBS). The specificities of the purified anti-EGFR chimeric mAbs were examined by ELISA (Fig 5A), which showed that both of the purified hIgG1 and mIgG3 chimeric mAbs were specific to EGFR (Fig 5A). Furthermore, the affinities of the chimeric mAbs were analyzed by surface plasmon resonance (SPR), and the calculated KDs of mIgG3 chimeric mAbs were almost identical to those of hIgG1 chimeric mAbs (Fig 5B).

### Generating mAbs genetically fused with fluorescent proteins

**Fig 6:**
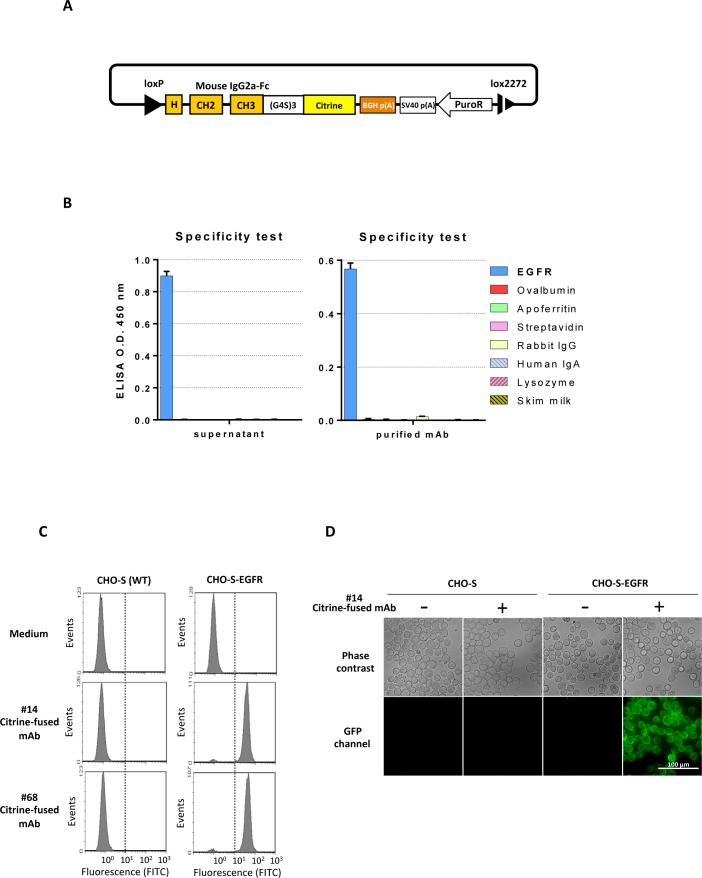
Generation of antibodies fused with a fluorescent protein. A) Design of pAbCitP donor vector to generate a Citrine-fused mouse chimeric mAb by RMCE. B) ELISA analysis to confirm the specificity after fusion of fluorescent protein. The culture supernatant (left) and purified antibody (right) were used. Negative control antigens are identical to those described in Fig 4B. C) Flow cytometry analysis of CHO-S cells. CHO-S cells overexpressing EGFR (right), and wild-type parental CHO-S cells (left), were incubated in the culture supernatant of the Citrine-fused anti-EGFR mAb expressing cells. Culture supernatants from two independent clones, #14 (middle) and #68 (lower) were examined. Culture medium of parental CHO-S was used as a negative control (upper). D) Fluorescence microscopy analysis using Citrine-fused mAbs. CHO-S cells overexpressing EGFR (right four panels) and parental CHO-S cells (four leftmost panels) were incubated in the culture supernatant of Citrine-fused anti-EGFR mAb-expressing cells and analyzed by fluorescence microscopy. The upper panels are phase contrast images and the lower panels are the images of GFP channel.

Antibodies labeled with fluorescent molecules are useful for many applications such as flow-cytometric analysis, fluorescence microscopy and *in vivo* imaging. We next generated a fluorescent antibody by fusing Citrine to the C-terminus of the antibody Fc region by RMCE [23]. Cells expressing hIgG1 chimeric anti-EGFR antibodies, which were isolated by the ADLib system, were co-transfected with a donor vector and Cre recombinase expression vector for RMCE. The donor vector named pAbCitP is composed of a mouse Fc region connected to Citrine by a (G_4_S)_3_ linker and a promoter-less puromycin resistance marker (Fig 6A). After puromycin selection, we confirmed successful replacement of Fc by the Citrine-fused Fc by detecting fluorescence from Citrine using flow cytometry (data not shown). ELISA analysis showed that the Citrine-fused antibody retained specific binding to EGFR, even after further purification with a Protein G affinity column (Fig 6B).

To assess the usefulness of the Citrine-fused antibody as a labeled antibody, CHO-S cells expressing human EGFR on their surface were incubated with the culture supernatant containing the Citrine-fused anti-EGFR antibodies. The CHO-S cells were subsequently analyzed by flow cytometry and fluorescence microscopy. Much stronger fluorescent signals were observed in EGFR overexpressing CHO-S cells compared to wild-type parental CHO-S cells after staining with the Citrine-fused antibodies (Fig 6C), and the fluorescence came from the Citritire-fused antibodies was enough to detect with a fluorescence microscope (Fig 6D).

Taken together, these data demonstrate that we can easily generate antibodies conjugated with fluorescence proteins by combining the ADLib system and RMCE, and the secreted antibodies in the culture supernatant are capable of binding to the target specifically while emitting sufficient fluorescence for cellular analyses.

### Generation of chimeric mAbs with enhanced affinity to FcγR

**Fig 7:**
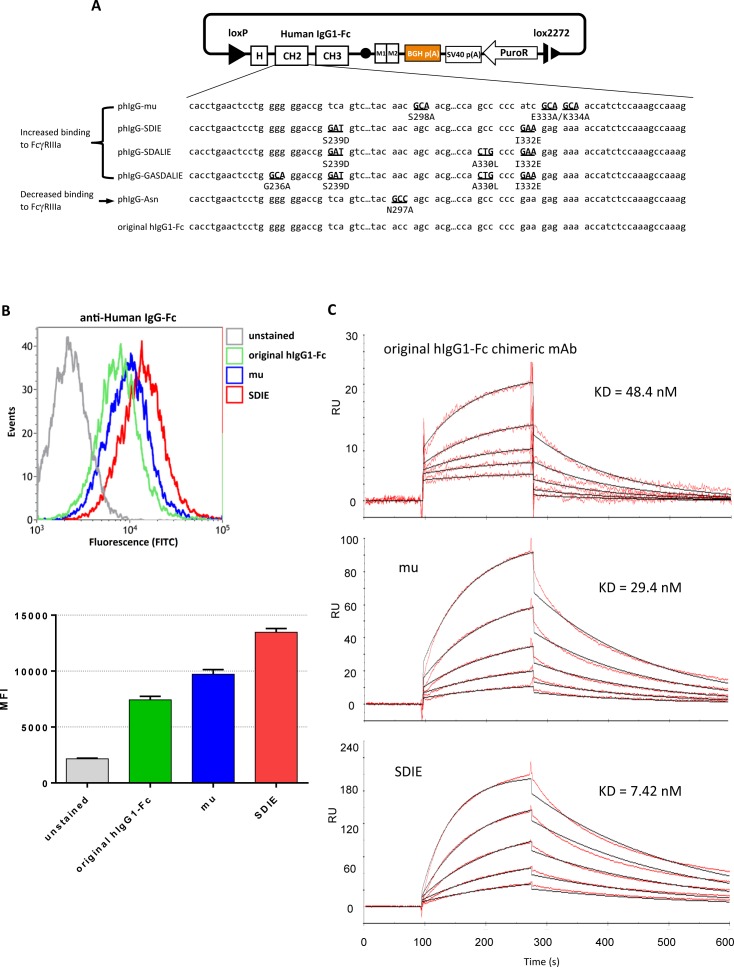
**Generation of Fc-mutated antibodies with enhanced affinity to Fc**γ**RIIIa** A) Design of donor vectors to generate hIgG1-Fc variants with mutated Fc, which were reported previously [36–38,44–46]. Four (phIgG-mu, phIgG-SDIE, phIgG-SDALIE, phIgG-GASDALIE) and one (phIgG-Asn) variants show increased and decreased binding to FcγRIIIa, respectively. DNA sequences and the amino acid substitutions of variants numbered in Kabat numbering are indicated on the right of the donor vector’s names. B) Flow-cytometric analysis examining the binding of variant Fc chimeric mAbs to FcγRIIIa. HEK293 cells overexpressing FcγRIIIa were incubated with each of the purified variant hIgG1-chimeric mAb. HIgG1 chimeric mAbs were detected by a FITC-conjugated anti-hIgG secondary antibody. The graph shows the mean fluorescence intensity (MFI) for each variant by n = 5 experiments. C) SPR analysis of variant hIgG1-Fc chimeric mAbs. Serially diluted hIgG1-Fc chimeric mAbs were applied to the FcγRIIIa immobilized biosensor chip. The KDs were calculated using 1:1 (Langmuir) binding model. FcγRIIIa has the allotypes at position 158 (Val or Phe), and we used FcγRIIIa (V158) in Fig 7B and 7C.

Fc engineering is useful to improve immunomodulatory functions such as antibody-dependent cellular cytotoxicity (ADCC), which depends on the affinities of Fcs to their receptors (FcγR) on the cell surface membranes. Previously, several hIgG1-Fc variants with either improved or impaired affinity to FcγR were reported [36–39]. To examine the applicability of our RMCE method to this Fc engineering, we next exchanged the Fc region of anti-EGFR hIgG1 chimeric mAb to its five variants. Four out of five variants were reported to show improved binding, and one displayed lower binding to FcγRIIIa [36–39] (Fig 7A). Cre recombinase expression vector and donor vectors containing each of the hIgG1-Fc variants were co-transfected (Fig 7A) and variant hIgG1 chimeric mAbs were generated. After RMCE, the specificity of each variant chimeric mAb to EGFR was confirmed by ELISA (S4A Fig).

To analyze the affinities of the variant hIgG1 chimeric mAbs to FcγRIIIa, we performed cell-based binding assay with flow cytometry and SPR. HEK293 cells overexpressing FcγRIIIa on their surface were incubated with variants of hIgG1 chimeric mAbs, and their binding to FcγRIIIa was analyzed by flow cytometry (Fig 7B). The relative binding intensity of each variant was consistent with the previously reported one [36,37]. The interactions of the variant hIgG1 chimeric mAbs with FcγRIIIa were further analyzed by SPR (Fig 7C and S4B Fig). The KD values were also consistent with those of previous reports [36–39]. These data suggest that RMCE can also be applied to the design of engineered Fcs with improved immunomodulatory functions.

## Discussion

We have demonstrated a rapid method to engineer the Fc regions of antibodies in DT40 cells by using RMCE. HIgG1 Fc coding sequence was knocked into the Fc region of DT40 IgM locus by gene targeting disrupting the Cμ2 exon of chicken IgM. The knock-in plasmid contains loxP and lox2272 sites so that 1) the hIgG1-Fc region can be replaced to any other sequences by Cre recombinase, and 2) only cells in which the Fc regions are properly rearranged at loxP and lox2272 sites are viable after co-transfection of the Cre recombinase expression vector and the donor vector for RMCE.

This strategy enhances the rapidity of our method because it enables the selection of successfully integrated cells by simple addition of antibiotics after transfection. This allows us to bypass the clonal expansion step to select recombinants, which is necessary for the conventional knock-in method [28]. Moreover, the short doubling time (8 hours) of DT40 cells also contributes to the rapid generation and expansion of targeted cells [28]. The minimal time required for generating a cell population with precise replacement of its Fc region is five days from the transfection of the donor vector. If the Fc region of DT40 cell is swapped by the conventional knock-in method, at least one week is needed for clonal expansion, and even more time is needed for the identification of positive clones by immunodot blot analysis or PCR. In addition, these screening steps are not easy due to the low success rate of gene targeting at the immunoglobulin locus of DT40.

The combination of this method with the ADLib system is also more rapid and powerful than other Fc engineering methods when considering the time required from the isolation of antigen-specific clones to the acquirement of desired chimeric mAbs. In contrast, with the current hybridoma method, the acquirement of chimeric mAbs would take several months, assuming the following engineering process: i) isolation of the mAbs’ heavy and light chain cDNAs; ii) generation of the chimeric heavy chain genes by assembly PCR; iii) cloning of the light chain and chimeric heavy chain cDNAs to the expression vectors; iv) transfection of the expression vectors prepared in iii) to cultured cells [40]. Moreover, the complexity of such Fc engineering makes it quite laborious to replace the Fc region of multiple hybridoma clones simultaneously. The isolation of the antigen-specific clones by the ADLib system in about a week and the following direct acquisition of Fc exchanged cells by transfection and drug selection in five days save considerable time and make it possible to handle multiple antigen-specific clones.

One of the key features of our method is the replacement of the native polyA terminator of chicken immunoglobulin heavy chain to the stronger BGH terminator (Fig 3). This enhances the expression of chimeric antibodies, which in turn enhances the efficiency of the ADLib system by increasing the expression level of mAbs on DT40 surfaces. Indeed, from the libraries constructed with hIgG1 knock-in cells, cells expressing antibodies specific to EGFR and lysozyme were successfully isolated using the ADLib system (Fig 4A and 4B). The hIgG1-Fcs of these cells were then switched to mouse Fcs by RMCE, and the resultant cells expressed mouse chimeric mAbs, while retaining their specificities to the antigens (Fig 4C and S2 Fig). These antigen-specific chimeric mAbs can be further purified by Protein G column without any major loss in affinities (Fig 5).

Here, we also demonstrate two tangible applications of our novel RMCE method for rapid Fc engineering. The first one was the generation of fluorescent protein-conjugated antibodies (Fig 6). The Fc region of anti-EGFR hIgG1 chimeric mAb was switched to Citrine-fused mIgG2a-Fc. This Citrine-fused mAb was readily applicable to flow cytometry and fluorescence microscopy without fluorescence-labeled secondary antibodies (Fig 6C and 6D). This suggests that various antibodies with direct applications as reagents can be prepared from our libraries. Theoretically, it is possible to prepare antibodies fused with proteins other than fluorescent protein. For example, fusion of primary antibodies with peroxidase or alkaline phosphatase [41] will provide useful tools for enzyme-linked immunological assays, suggesting further applications to diagnostic purposes.

Fc engineering has also been an important topic in the field of antibody therapeutics, since the Fc region plays a pivotal role in major immune reactions such as ADCC response [42,43]. It has been reported that ADCC activity can be enhanced by introducing amino acid substitutions into Fc or by removal of the fucose residue from oligosaccharides linked to Fc Asn297 [36–38,44–46]. In this study, we demonstrated that our RMCE method enables the generation of chimeric mAbs with hIgG1-Fc variants that harbor previously reported amino acid substitutions conferring enhanced binding to FcγR, and the obtained chimeric mAbs showed improved affinity to FcγRIIIa, consistent with previous literature (Fig 7). To develop such novel engineered Fcs, various candidates have to be tested, which is laborious by conventional methods. Therefore, our rapid method to exchange Fc region will accelerate the development of engineered Fcs with novel functions.

The method in this study enables functionality enhancement of antibodies generated by the ADLib system. By rapidly exchanging their Fcs to the various engineered ones, it will be possible to select the most suitable engineered Fc that confers the best efficacy or add-on function of interest to the user. Moreover, if engineered Fcs with novel functions are developed in the future, our method will enable the immediate testing of them by generating chimeric mAbs rapidly. The versatility of this method will contribute to generate more effective mAbs for therapeutic, diagnostic or research purposes.

## Materials and Methods

### Cell culture

DT40 cells were cultured in a CO_2_ incubator at 39.5°C in IMDM, GlutaMAX Supplement (Invitrogen) supplemented with 10% FBS, 1% chicken serum, penicillin, streptomycin, and 2-Mercaptethanol. Culture media was changed regularly and cell density was maintained below 2.0 x 10^6^ cells/mL. To prepare the DT40 cell based libraries for the ADLib system, DT40 cells were cultured in medium supplemented with TSA (Wako) at the concentration of 2.5 ng/mL. For the purification of chimeric IgG, 2.0 x 10^6^ cells/mL DT40 cells were cultured in serum free AIM-V (Invitrogen) for 48 hours. CHO-S cells were cultured in a CO_2_ incubator at 37°C in CHO-S-SFM II (Invitrogen). CHO-S cells expressing human EGFR, provided by Chiome Bioscience Inc., were cultured in the medium supplemented with G418. HEK293 cells were cultured in a CO_2_ incubator at 37°C in DMEM, high glucose, GlutaMAX (Invitrogen) supplemented with 10% FBS, penicillin and streptomycin. For establishing HEK293 cells expressing human Fc©RIIIa, the cDNA of human FcγRIIIa (available from the Riken DNA Bank (clone ID: IRAK049H17)) was cloned into pIRESneo (Clontech) based expression vector of which neomycin resistance gene was replaced with puromycin resistance gene and transfected to HEK293 cells by HilyMax (Dojindo).

### Plasmid Constructs

In the previous work, we reported a targeting vector to insert exogenous sequences into the intronic region between exon Cμ1 and Cμ2 of chicken IgM. To construct p2.2-hIgG1, we modified the right arm sequence for target integration to disrupt exon Cμ2 of chicken IgM. In p2.2-hIgG1, the synthetic sequence of hIgG1-Fc, which lacks the intron sequence between exon M1 and M2 for membrane-bound domain, was placed downstream of the loxP sequence, and a puromycin resistance marker, which has the lox2272 sequence downstream of the CMV promoter, was inserted downstream of the hIgG1-Fc in the opposite orientation. The Cre recombinase expression vector pCAG-Cre consists of the CAG promoter, which is expected to induce strong expression, and the Cre recombinase gene. The donor vector pmG2A-4 was constructed from pcDNA6/myc-His B (Invitrogen). The sequences from the CMV promoter to the SV40 polyA signals were substituted with synthetic sequences that consist of the loxP sequence, mIgG2a-Fc amplified from mouse genomic DNA, a promoter-less blasticidin S resistance marker placed in the opposite orientation and the lox2272 sequence. The donor vector phIgG-3 was composed of the loxP sequence, hIgG1-Fc amplified from human genomic DNA, a promoter-less blasticidin S resistance marker placed in the opposite orientation and the lox2272 sequence. To increase the expression level of the chimeric mAb, the native terminator sequence downstream of exon M2 was replaced with BGH terminator sequence in phIgG-3. The donor vector pmG2A-4BP was prepared from pmG2A-4 by exchanging the native terminator sequence downstream of exon M2 with the BGH terminator sequence and replacing the blasticidin S resistance marker with a puromycin resistance marker. The donor vector pmG3-4BP was prepared from pmG2A-4BP by replacing mIgG2a-Fc with mIgG3-Fc amplified from mouse genomic DNA. The donor vector pAbCitP was constructed from pcDNA6/myc-His B (Invitrogen). The sequences from the CMV promoter to the SV40 polyA signals were removed from the original plasmid as was done with pmG2A-4, and substituted with synthetic sequences that consist of the loxP sequence, mIgG2a-Fc linked to the fluorescent protein Citrine with a flexible linker (G_4_S)_3_, a promoter-less puromycin resistance marker placed in the opposite orientation and the lox2272 sequence. The donor vectors phIgG-mu, phIgG-SDIE, phIgG-SDALIE, phIgG-GASDALIE, phIgG-Asn were prepared from pmG2A-4BP by replacing mIgG2a-Fc with mutated hIgG1-Fc. Site-directed mutagenesis was performed by PCR using mutated primers.

### Transfection of p2.2-hIgG1 plasmid and isolation of the positive clones

40 μg of p2.2-hIgG1 plasmid was linearized with BglII to transfect 1.0 x 10^7^ wild-type DT40 cells expressing IgMs by electroporation (Bio-Rad Gene Pulser, 0.4 cm gap, 550 V, 25 μFD). After 24 hours of incubation at 37°C, stable clones were selected in 96-well plates with a medium containing 0.25 μg/mL puromycin (Invitrogen). Positive clones expressing hIgG1s were screened by immunodot blot analysis.

### Cassette replacement by RMCE

Nucleofector Technology (Lonza) was used for RMCE. 1.0 x 10^7^ DT40 cells were suspended with the provided solutionT, and 0.8 μg of Cre recombinase expression plasmid and 7.2 μg of the donor plasmid were co-transfected by nucleofection using Program B-23. After 24 hours of incubation at 39.5°C, antibiotics were added to the culture medium and incubated for another four days to select cassette-replaced DT40 populations.

### Immunodot blot assay

2 μL of culture supernatant was spotted onto a nitrocellulose membrane (GE Healthcare). After blocked by 5% skim milk in TBST, the membrane was incubated with HRP-conjugated secondary antibodies (either anti-chicken IgM-Fc (Acris, 1:50,000 dilution), anti-hIgG-Fc (Sigma, 1:2,000 dilution) or anti-mIgG-Fc (Bethyl, 1:1,000 dilution)). Signals were detected using the ECL Western Blotting Analysis System (GE Healthcare).

### Flow cytometry (FACS)

1 x 10^6^ DT40 cells were washed with FACS buffer (2 mM EDTA- 0.5% BSA-PBS). Cells were suspended with FITC-conjugated secondary antibodies (either anti-chicken IgM-Fc (MYBioSource, 1:50 dilution), anti-hIgG-Fc (Bethyl, 1:200 dilution) or anti-mIgG-Fc (Bethyl, 1:200 dilution)) diluted in FACS buffer and incubated for 15 min at 4°C. Stained cells were washed with FACS buffer and analyzed by FC500 flow cytometer (Beckman Coulter).

### RT-PCR analysis

Total RNA was extracted from DT40 cells with TRIzol reagent (Invitrogen) and reverse transcription was performed with Prime Script II 1st strand cDNA synthesis Kit (Takara). CDNA was amplified by PCR with the following thermal-cycling procedure; 94°C for 2 min, followed by 30 cycles of 98°C for 10 sec and 68°C 1.5 min. The sequence information of primers is described in S5 Fig.

### Real-time quantitative RT-PCR

Reverse transcription was performed with Prime Script RT reagent Kit (Perfect Real Time) (Takara). Real-time RT-PCR was performed with SYBR Premix ExTaqII (Tli RNaseH Plus) (Takara) using the StepOnePlus System (Thermo Fisher Scientific). Sequence information of primers is described in S5 Fig.

### ELISA

ELISA was performed as described [7,27]. Briefly, maxisorp immunoplates (Nunc) were incubated overnight at 4°C with the antigen diluted at 3 μg/mL in PBS. After blocking with 1% BSA in PBS for 30 min at room temperature and washing three times with PBST, culture supernatants or purified mAbs were added to the plates and incubated for 1 hour at room temperature. After washing five times with PBST, HRP-conjugated secondary antibody (either anti-chicken IgM-Fc (Bethyl, 1:10,000 dilution), anti-hIgG-Fc (Bethyl, 1:5,000 dilution) or anti-mIgG-Fc (Bethyl, 1:5,000 dilution)) solution was added to the plates and incubated for 1 hour at room temperature. After washing the plates five times with PBST, 3,3’,5,5’-tetramethylbenzidine (TMB) (Dako Cytomation) was added, and the reaction was stopped with 1 N sulfuric acid. The optical density at 450 nm was measured with a microplate reader Model 680 (Bio-Rad). In the specificity test, each analysis was examined by n = 5 experiments.

### mAb selection with the ADLib system

The details of the ADLib system were previously reported [7,27]. Briefly, for magnetic beads selection, antigens were conjugated with Dynabeads M-280 Tosylactivated. Then antigen-coated magnetic beads and DT40 cell based libraries were mixed in selection buffer (1% BSA in PBS) and incubated for 30 min at 4°C, gently rotating. After washing three times with selection buffer using a magnetic stand, the recovered cells were diluted with fresh medium, dispensed into 96 well plates and incubated for 1 week in a CO_2_ incubator at 39.5°C. DT40 cells expressing the antigen-specific antibodies were screened by ELISA.

### Antibody purification

Chimeric antibodies were purified by AKTApurifier using HiTrap Protein G HP columns (GE Healthcare). Binding, elution and neutralizing buffers were prepared as per the manufacturer’s instructions.

### Surface plasmon resonance (SPR) analysis

SPR analysis was performed by Biacore 2000 (GE Healthcare). Recombinant human EGFR-Fc chimera (R and D systems) in sodium phosphate pH 5.5 at 10 μg/mL was immobilized on the surface of a CM5 biosensor chip using amine coupling chemistry which resulted in a surface density of approximately 10,000 RU. For kinetic analysis, purified mAbs serially diluted with running buffer (1% BSA-HBS-EP) were injected at a flow rate of 20 μL/min. The regeneration was carried out by injecting 1 M MgCl_2_ for 1 min. Data were fitted with BIAevaluation software (GE Healthcare) on the basis of a bivalent analyte model. As to the mutated hIgG1-Fc chimeric mAbs, recombinant human FcμRIIIa (R and D systems) in sodium phosphate pH 5.5 at 5 μg/mL was immobilized on the surface of a CM5 biosensor chip using amine coupling chemistry which resulted in a surface density of approximately 1,000 RU. For kinetic analysis, hIgG1-Fc variants with mutated Fc serially diluted with running buffer (1% BSA-HBS-EP) were injected at a flow rate of 20 μL/min. The regeneration was carried out by injecting 1 M MgCl_2_ for 1 min. Data were fitted with BIAevaluation software on the basis of a 1:1 (Langmuir) binding model.

## Supporting Information

S1 Fig Screening results of hIgG1 knocked in cells by immunodot blot analysis with a HRP-conjugated anti-hIgG-Fc antibody. The culture supernatants of clones that were refractory to puromycin after transfection of p2.2-hIgG1 were examined to identify positive clones in which the IgM-Fc region was replaced with the relevant sequence in the targeting vector. Spots surrounded by red lines correspond to 220 representative clones among 261 puromycin-resistant candidates.
(EPS)

S2 Fig ELISA analysis to confirm the specificities of mIgG2a chimeric mAbs. After the antibody selection against EGFR and lysozyme by the ADLib system, the Fc region was replaced with mIgG2a by RMCE. Specificities of anti-EGFR (left) and anti-lysozyme (right) mAbs were examined by ELISA. Negative control antigens are identical to those described in Fig 4B.
(EPS)

S3 Fig Isolation of antigen-specific mIgG2a chimeric mAbs by the ADLib system. A) Screening of mIgG2a chimeric mAbs specific to EGFR by ELISA. MIgG2a chimeric mAb-expressing cells are prepared by RMCE. Libraries constructed from these cells were subjected to the ADLib system by using magnetic beads conjugated with EGFR. The culture supernatants of the selected clones were analyzed by ELISA to examine the binding activity to EGFR. Ovalbumin was used as a negative control. The clones indicated by the arrows were further analyzed in the experiment described in S3B Fig. B) ELISA analysis to confirm the specificities of the culture supernatants of mIgG2a chimeric mAbs. The clones used are indicated by the arrows in S3A Fig. Negative control antigens are identical to those described in Fig 4B.
(EPS)

S4 Fig Specificities and binding kinetics of Fc-mutated chimeric mAbs with modified affinities to FcγRIIIa. A) ELISA analysis to confirm the specificities of the variant hIgG1-Fc chimeric mAbs. The Fc region of the anti-EGFR hIgG1 chimeric mAb was replaced with each variant hIgG1-Fc by RMCE. The specificities of the culture supernatants against EGFR were examined by ELISA. Negative control antigens are identical to those described in Fig 4B. B) SPR analysis of variant hIgG1-Fc chimeric mAbs. Serially diluted chimeric mAbs were applied to the FcγRIIIa immobilized biosensor chip. The KDs were calculated using 1:1 (Langmuir) binding model.
(EPS)

S5 Fig Primers for RT-PCR and real-time quantitative RT-PCR amplification. (EPS)
